# American Academy of Dental Surgery

**Published:** 1876-11

**Authors:** 


### ARTICLE IV.

### *American Academy of Dental Surgery.*

Reported for the American Journal of Dental Science.

The regular meeting of the Academy was held on Tuesday evening, the 26th September. President, Dr. Geo. H. Perine, in the chair.

The minutes of the special and regular meeting, held in Philadelphia, on the 25th of July, were read and approved. Donations to the library and museum were received, and a vote of thanks returned

Dr. Geo. E. Meigs, on behalf of the Executive Committee, presented the application of active and correspond ing fellowship of a number of gentleman who were unanimously elected. The subject of the meeting—"Dental education with that of medicine, by the appointment of dental chairs and professorships in medical colleges and dental practice in hospitals," which has been agitated for some time past by the Academy was fully discussed. The President gave a resume of the action of the meeting held in Philadelphia, in July last. The Secretary, Dr. C. D. Hayward, read letters approving of the action of the Academy, and their desire to aid in forwarding its objects: From T. Davis Fitch, M. D., President of the Illinois State Medical Society; Dr. E. L. Hunter, Secretary of the North Carolina State Dental Association; Prof. Joseph Leidy, M. D., University of Pennsylvania, and Prof. Lear tus Connor, M. D., Detroit Medical College. A resolution was presented and adopted, recommending to the medical body the establishment of dental professorships in medical colleges. A committee was appointed to take the matter in hand and forward the object as speedily as possible, and communicate with the medical Faculty, and report at the next meeting.

A paper by Corresponding Fellow, Dr. Wm. C. Eastlacke, Berlin, Prussia, was read, in which special reference was made to the combination of oxychloride in combination with gold. The writer thought in some cases it was safer and better than solid gold fillings, however, well made; you can pack over these fillings, which when carefully done, will be as good as all gold fillings. He has followed this line of practice for several years, and has observed the teeth so treated in better condition than those filled entirely of gold, however well made. How many of us can recall cases where the death of the pulps has been occasioned by thermal changes. Here we have the preventive; if good in one case, why not always, reierring to those teeth that are advanced in decay, not to small superficial cavities. He pronounced it a good capping for exposed pulps and admissable as a temporary filling. Describing his *modus operandi*, after the tooth has been prepared and the rubber dam applied, he introduces the oxychloride, waiting sufficient length of time for it to harden, he trims to the required depth and prepares its surface for the gold filling, filling the cavity thus prepared with "Hill's Stopping," reserving another sitting for the completion of the operation, at which time he removes the gutta percha, finds the oxychloride hard and perfectly free from moisture, —the gold filling is then made. He thinks the advantage by filling badly decayed front teeth in this way are incalculable. Their natural color is preserved, their delicate walls are strengthened, and they are almost entirely free from pain, caused by the sudden changes of temperature to which these teeth are constantly exposed. The writer gave a record of many cases thus successfully treated, proving incontrovertable the value of this article employed in the manner he described. The subject was freely discussed by the Fellows present, various opinions were expressed and practical experience was given.

Dr. Perry, invited attention to the method introduced to the profession by Dr. Geo. H. Perine, in an illustrated article on "Filling Teeth," which appeared in the March number, (page 515,) of The American Journal of Dental Science.

By invitation, Corresponding Fellow, Dr. W. O. F. Bascome, of Bermuda, followed in an address, giving a comprehensive description of the advancement of the profession in South America and the West Indies.

The President announced that J. D. Miles, M. D., of Philadelphia, would read a paper at the next meeting, which will occur on the fourth Tuesday in October, when the meeting adjourned.

